# Curcumin Electrochemistry—Antioxidant Activity Assessment, Voltammetric Behavior and Quantitative Determination, Applications as Electrode Modifier

**DOI:** 10.3390/antiox12111908

**Published:** 2023-10-25

**Authors:** Iulia Gabriela David, Emilia Elena Iorgulescu, Dana Elena Popa, Mihaela Buleandra, Mihaela Carmen Cheregi, Hassan Noor

**Affiliations:** 1 Department of Analytical Chemistry and Physical Chemistry, Faculty of Chemistry, University of Bucharest, Panduri Av. 90-92, District 5, 050663 Bucharest, Romania; elena.popa@chimie.unibuc.ro (D.E.P.); mihaela.buleandra@g.unibuc.ro (M.B.); mihaela.cheregi@g.unibuc.ro (M.C.C.); 2 Department of Surgery, Faculty of Medicine, “Lucian Blaga” University Sibiu, Lucian Blaga Street 25, 550169 Sibiu, Romania; hassan.noor@ulbsibiu.ro; 3 Medlife-Polisano Hospital, Strada Izvorului 1A, 550172 Sibiu, Romania

**Keywords:** curcumin, voltammetry, electrochemistry, antioxidant, anticancer, curcumin complexes

## Abstract

Curcumin (CU) is a polyphenolic compound extracted from turmeric, a well-known dietary spice. Since it has been shown that CU exerts beneficial effects on human health, interest has increased in its use but also in its analysis in different matrices. CU has an antioxidant character and is electroactive due to the presence of phenolic groups in its molecule. This paper reviews the data reported in the literature regarding the use of electrochemical techniques for the assessment of CU antioxidant activity and the investigation of the voltammetric behavior at different electrodes of free or loaded CU on various carriers. The performance characteristics and the analytical applications of the electrochemical methods developed for CU analysis are compared and critically discussed. Examples of voltammetric investigations of CU interaction with different metallic ions or of CU or CU complexes with DNA as well as the CU applications as electrode modifiers for the enhanced detection of various chemical species are also shown.

## 1. Introduction

### 1.1. Curcumin—History and Occurrence

Turmeric was used 4000 years ago in cuisine and traditional medicine in India and China [1], but curcumin (CU) as its main component was discovered in turmeric only in 1815 and obtained as a pure compound in 1842. Its chemical structure and its synthesis were reported in 1910 and 1913, respectively [2]. Nowadays, it is mainly cultivated in India and China, but also in other tropical regions from South Asia, Africa, South America [3], and the Pacific basin [4].

**Figure 1:**
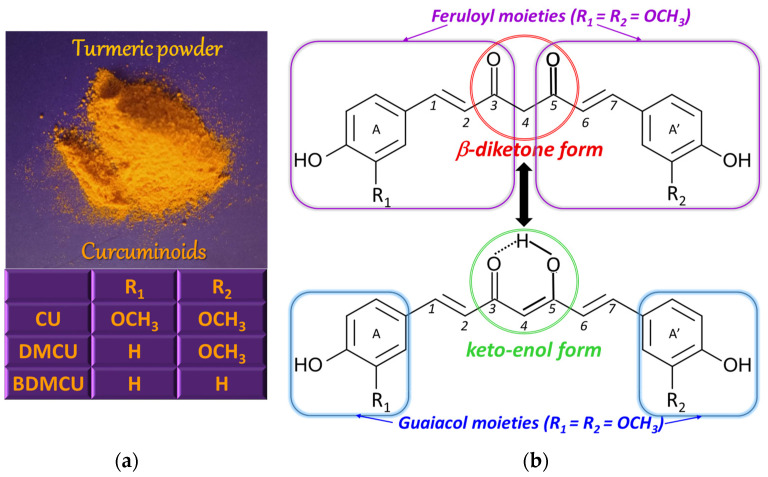
Curcuminoids found in turmeric (**a**) and their tautomeric equilibrium (**b**).

CU [(1*E*,6*E*)−1,7−bis(4−hydroxy−3-methoxyphenyl)hepta−1,6−diene−3,5−dione] or diferuloylmethane [5] (Figure 1) is a solid polyphenolic antioxidant with bitter taste [6], extracted from the rhizome of the perennial herb turmeric (*Curcuma longa* Linnaeus), but also from other plants of the ginger (*Zingiberaceae*) family, where it coexists with structurally related species, known under the collective name of curcuminoids [5]. The main curcuminoids are CU, demethoxycurcumin (DMCU), and bis-demethoxycurcumin (BDMCU) (Figure 1). They differ by the number of methoxy groups on the aromatic nuclei. All of them are bioactive, possessing antioxidant properties, but, among them, CU is the most effective, having higher antioxidant power than vitamins C and E [3].

The amount of curcuminoids (3–5%) [7]) in turmeric roots depends on the growing conditions, including soil type and climate [5], while the CU content of commercially available turmeric powder varies in the range 0.5–5.7%, also being influenced by the harvesting, extraction, and processing procedures [8]. Despite the fact that the literature reported somewhat different proportions of CU, DMCU, and BMCU, namely approximately 2:1:1 [8], 75%, 25%, and 5% [9] and 70%, 20%, and 10% [7], respectively, the major component of turmeric rhizome is CU.

### 1.2. Curcumin—Chemical Structure and Properties

From the chemical point of view, the CU molecule is composed of two ferulic acid molecules (feruloyl moieties) linked via a methylene group [10]. Going into more detail, the CU structure consists of two ortho-methoxy phenolic (guaiacol) groups bridged by a seven carbon atoms chain that contains an α,β-unsaturated β-diketone moiety, which determines the keto-enol tautomerism (Figure 1) [8], the enol proton being evenly distributed between the two oxygen atoms due to the symmetry of the CU molecule [11]. The ratio of the two tautomeric forms in solution depends on the solvent and its polarity, the solution pH, and temperature [7]. The keto form exists in acidic and neutral solutions (about 70%) [12] and in cell membranes, while the enol form is preponderant in alkaline media [9], in ethanol [8], in nonpolar organic solvents, and in solid phase, being stabilized by hydrogen bonds [10]. The CU biological properties are due to the guaiacol moieties as well as the keto-enol site [11].

CU undergoes three acid–base equilibria, the first one with pK_a1_ values reported in the range 7.43–8.55 and involving the transfer of the proton from the enol group. The next dissociation steps, attributed to the deprotonation of the two phenolic –OH moieties, have relatively close pK_a_ values (pK_a2_ in the range 8.55–10.45 and pK_a3_ varying between 9.05 and 10.95) due to the symmetric position of the corresponding protogenic groups in the CU molecule [13].

Initial studies reported that CU is stable in solutions with pH values below 7.00, its dissociation equilibrium being shifted towards the neutral form, with poor aqueous solubility when the environment becomes more acidic. CU instability in alkaline media was explained by its hydrolytic degradation [14]. After CV and spectrophotometric investigations of CU behavior in time at different pH values, Martínez-Guerra et al. [13] concluded that CU degradation is 20 times faster in acidic media than in neutral or basic solutions, but CU stability in aqueous environments can be improved by deaerating and protecting the solution from light.

CU is practically insoluble in acidic and neutral aqueous solutions and poorly soluble in hydrocarbon solvents [15], but it is soluble in alkali [14], in lipids [10], and in organic solvents like acetic acid, ethanol [7], methanol, DMSO [12], acetone, and dichloromethane, its extraction in acetone being more efficient than in ethanol [16].

### 1.3. Curcumin—Uses

CU popularity increased remarkably worldwide in recent years as a consequence of its nutritional, prophylactic, and therapeutic values. Due to its orange–yellow color, it found applications as a natural coloring agent for food (mustard, margarine, processed cheese [17], pastries, canned products, and beverages [18]), cosmetics, hair dyes [19], textiles, furniture, and lacquers [20]. CU can also legally be added to food [21] as a preservative, spice, flavoring agent [22], and antioxidant in dairy products, meat, and seafood (fish, shrimp) [7]. The CU content of foods varies between 5 mg/kg and 500 mg/kg [23]. Moreover, on the market, there is a large variety of nutraceuticals and dietary supplements containing turmeric or its most bioactive component, CU, which are consumed on a large scale by the population [10]. A daily dose of 12,000 mg CU, which corresponds to a concentration of 51.2 ng/mL in human serum [23], presented little to no side effects, and, therefore, the FDA classified it as GRAS [24], the daily consumption level approved by the WHO being 1–3 mg/kg body weight [4,6]. At higher concentrations and prolonged administration, CU can inhibit the activity of some enzymes and cause anemia in persons with reduced iron uptake or various gastrointestinal [25], liver, inflammatory, and anticoagulation [12] problems.

Photoactivated CU encapsulated in β-CD was used for the antibacterial treatment of berries, increasing their shelf life without changing their organoleptic properties [26], while recent studies emphasized that CU-NPs, even at low concentrations, improved soybean growth and could be employed as fertilizer [27]. CU loaded into a zein/shellac-based composite food packaging film conferred antioxidant properties, inhibited *E. coli*, and the color change with pH variation enabled the monitoring of the food freshness [28]. There are many such applications of CU in the development of bioactive thin-layer composite polymeric food packaging films, and they were recently reviewed by Roy et al. [7].

CU also has beneficial effects on the growth of chickens and their egg production, curcuma being used as a feed additive in the broiler poultry industry [29].

Due to its lipophilic character, CU can bind to the amyloid β-oligomers that generate brain dysfunctions [30], and, therefore, it presents a dose-dependent enhancement of learning ability and memory by leading to beneficial results in the treatment of Alzheimer’s disease, both alone or in combination with coenzyme Q10 [31] or in the combined treatment of Fabry disease [24]. It has cardioprotective effects [32], reduces inflammation in patients with chronic renal failure [33] or in patients recovered from COVID-19 [34], may prevent and treat liver injury caused by aflatoxin B1 [35] or its age-related senescence [36], has therapeutic effects on hyperglycemia, oxidative stress, kidney, and nonalcoholic fatty liver diseases induced by high fat diet [37,38], reduces muscle damage and inflammation and improves sport performances [39], can protect human or animal muscles from degeneration [40,41], the lungs against air-pollution-induced inflammation [42], and the skin against UV radiation, having antimelanogenic [43] and wound-healing properties [44], also being employed as an active ingredient in cosmetic products [45]. Due to its antioxidant and metal-chelating properties, CU could be a treatment for metal poisoning [10]. It was shown that CU at concentrations of 5.00 × 10^−6^–5.00 × 10^−5^ mol/L at the cancer cell level [46] has antitumoral effects [5,47]; for example, it inhibits the proliferation of breast cancer cells [48] and exerts a dose-dependent reduction in the growth and progression of adrenocortical carcinoma [49], prostate cancer [50], rhabdomyosarcoma [51], colorectal [52,53], and bladder tumoral cells [54]. Moreover, at high concentrations, it has pro-oxidant properties, generating intracellular ROS that induced apoptosis of human lung cancer cells resistant to docetaxel and vincristine [55]. Depending on its concentration, CU exerts an anti- or pro-oxidant effect on DNA [14,56]. Among the CU pro-health activities are also the antibacterial effect discovered in 1949 [12,15], antiviral (against HIV, HPV, hepatitis virus, etc.) [57], and immunomodulatory and potentially antiallergic ones [1].

**Figure 2:**
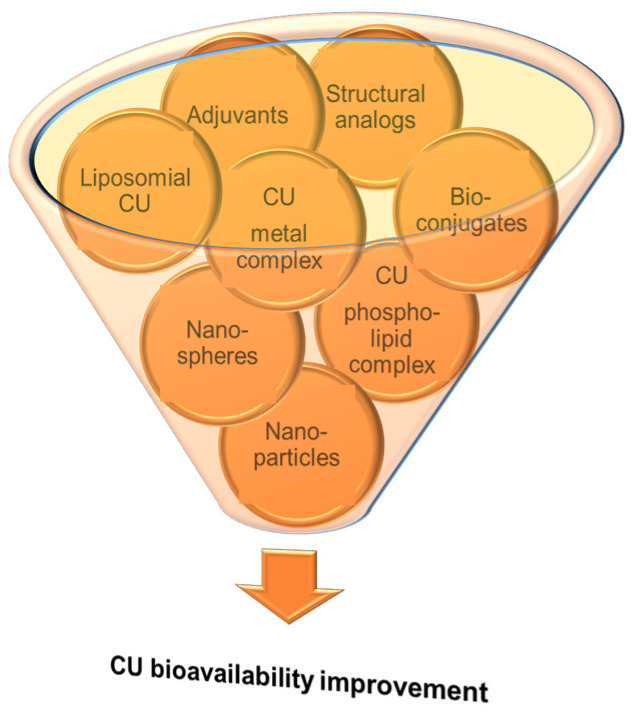
Different methodologies used to enhance the bioavailability of curcumin (adapted after [10]).

Despite its many health benefits, which were also discussed in different sections of some reviews [10,12,58,59], CU therapeutic use is limited by its reduced bioavailability generated by its low aqueous solubility, poor intestinal absorption, rapid metabolism and excretion from the body, 75% of the administrated CU dose being found in animal feces [10]. The highest CU concentration is in the intestine, while, in plasma or other tissues, it can be smaller than the quantification limits of the commonly applied analytical methods. In plasma, CU could be detected only after oral administration of high doses (at g levels), the maximum plasmatic level being reached 1–2 h after ingestion [58]. That is why researchers are continuously concerned to enhance the stability and bioavailability of CU in various ways (Figure 2), some of them being summarized in different review papers [9,10,12,15,58,60,61,62].

There are also reports presenting an interesting application of CU and its derivatives as a corrosion inhibitor [63,64].

The multiple different applications of CU, generated by its various beneficial functional properties, have increased its demand worldwide in recent years, and this trend continues such that the global CU market was estimated to be USD 104.19 million in 2025 [7]. However, CU’s main use still remains in the food and dietary supplements industry.

Despite the fact that CU ingestion presents a high degree of safety for animals, considering its concentration-dependent anti- and pro-oxidant activity towards DNA and also other possible side effects that could appear after the administration of high CU doses, it is important to have simple and rapid methods for its sensitive and selective quantification in foods and dietary supplements, as well as in biological samples. The recent literature includes reports related to the spectrometric [65,66,67,68], fluorimetric [57,69,70,71], chromatographic [72,73,74,75], and electrochemical [19,23,76,77,78] analysis of CU. Analytical methods applied to curcuminoids assessment in turmeric, including CU, were reviewed in 2019 by Kotra et al. [79], with special emphasis on the chromatographic and spectrometric ones, electrochemical methods being very briefly mentioned. A few examples of voltammetric determination of CU in spices were discussed in 2018 by Ziyatidinova and Budnikov [80] in a review paper related to the analytical chemistry of spice antioxidants. The examples of CU electrochemical detection presented in 2019 by Mohajeri et al. [81] in a synthesizing article that addressed the interaction between CU and carbon-based nanomaterials were limited only to the biosensors using this type of sensing material. In a recent review on the CU extraction and analysis procedures, electrochemical methods and sensors are mentioned [12]. Each of the analytical methods have certain advantages and drawbacks, but the electrochemical ones are simpler, user-friendly, more rapid, involve fewer reagents (thus being eco-friendly), and, most importantly, voltammetry allows the explanation of certain reaction mechanisms that are the basis of some biological activities, such as antioxidant activity.

Therefore, based on the data published in the specialized literature in the last 20 years, this review discusses in detail the role of the electrochemical sensors and methods in investigating diverse aspects of CU analysis, including not only its quantification in various matrices but also its redox behavior and some of its biological activities, especially the antioxidant and antitumoral ones. In addition, CU interaction with various chemical species and its application in the development of electrochemical sensors for the assessment of different analytes were addressed.

## 2. CU Electrochemical Behavior

Almost all papers published in the last 20 years related to CU voltammetric analysis tackled its electrochemical behavior at solid electrodes and exploited the oxidation signal(s) in the development of analytical methods for its quantification. There are only a few relatively old reports discussing CU electro-reduction, mainly at mercury electrodes. This last aspect is understandable considering that mercury electrodes are best fitted for the investigation of cathodic processes, but the use of these electrochemical sensing devices has been greatly reduced in recent years due to the toxicity of mercury.

### 2.1. Curcumin Electro-Reduction

CU electro-reduction was investigated by both polarography and voltammetry at mercury electrodes, and, although there are few reports, the proposed mechanisms are not quite unified.

**Figure 3:**
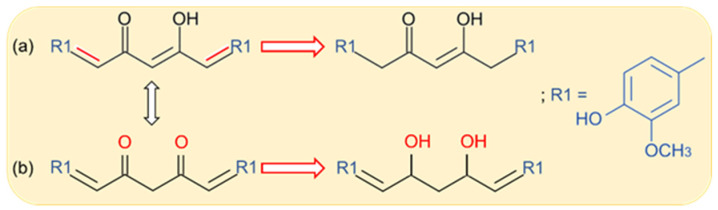
Possible CU electro-reduction mechanisms involving (**a**) each of the two double bonds situated next to the ketone and hydroxyl groups, and (**b**) the diketone group.

In 1.00 mol/L ammonium tartrate solution pH 8.10, CU existed in its enolic form, and it was shown that, at the DME, each of the two double bonds situated next to the ketone and hydroxyl groups, respectively, was reduced involving two electrons and two protons (Figure 3a), resulting in tetrahydrocurcumin. In the direct current and differential pulse polarographic curves, these processes generated a signal with the half-wave potential of −1.275 V and two cathodic peaks with the potentials E_pc1_ = −1.125 V and E_pc2_ = −1.275 V versus SCE, respectively. Due to its higher sensitivity, DPP was applied to quantify CU in turmeric powder and pharmaceutical formulations [82].

A CV investigation carried out at HMDE in BRB solution containing 1% ethanol pointed out that, in acidic medium (pH 6.00), CU presented two irreversible pH dependent cathodic peaks (E_pc1_ = ~−1.000 V and E_pc2_ = ~−1.200 V versus Ag/AgCl) generated by the reduction of the diketone group (Figure 3b), while, in a basic environment (pH 9.50), only one peak (E_pc_ = ~−1.100 V versus Ag/AgCl) was observed. Similar results were obtained by DPV [83].

In another study, three cathodic signals (E_pc2_ = −1.100 V, E_pc1_ = −1.300 V, and E_pc3_ = −1.600 V versus Ag/AgCl) were recorded for CU at HMDE in PBS (pH 8.50) + 0.10 mol/L NaCl after a previous anodic scan during which two small ill-defined anodic peaks (E_pa1_ = −1.300 V and E_pa2_ = −1.000 V versus Ag/AgCl) were observed. The authors considered that the cathodic peaks were probably the result of the keto moieties reduction. This is the only study that also reported the effect of the analyte concentration on the cathodic peak (E_pc_ = 0.300 V versus Ag/AgCl) corresponding to the reversible redox pair presented by CU at CPE [20]; all other research using solid electrodes, which will be discussed further, considered only one of the CU anodic peaks, either the reversible or the irreversible one (situated at more positive potentials).

### 2.2. Curcumin Electro-Oxidation

There are many reports briefly presenting [14,16,84,85,86,87] or discussing in detail [23,46,88,89,90,91,92,93,94,95,96,97,98,99,100,101,102,103,104,105] CU electro-oxidation mechanisms at various working electrodes. The voltammetric behavior of CU at solid electrodes was studied mainly in aqueous medium [14,23,85,88,89,90] at pH < 8.00 due to the fact that, in alkaline solutions, CU was considered to be unstable and underwent hydrolytic degradation to ferulic acid and feruloylmethane [16,18,91], but the results were similar in the case of some organic solvents [92,93,94]. The technique most often used for this purpose was CV, but other electrochemical methods such as DPV [93], SWV [95], chronoamperometry [18,96], and chronocoulometry [19,97] were also applied to confirm or complete the CV information. In order to establish a reaction mechanism as accurate as possible, in certain cases, in addition to CU, the voltammetric behavior of other structurally related compounds, such as ferulic acid, capsaicin and dihydrocapsaicin [95], BDMCU [92], or DMCU and BDMCU [98], was also studied.

**Figure 4:**
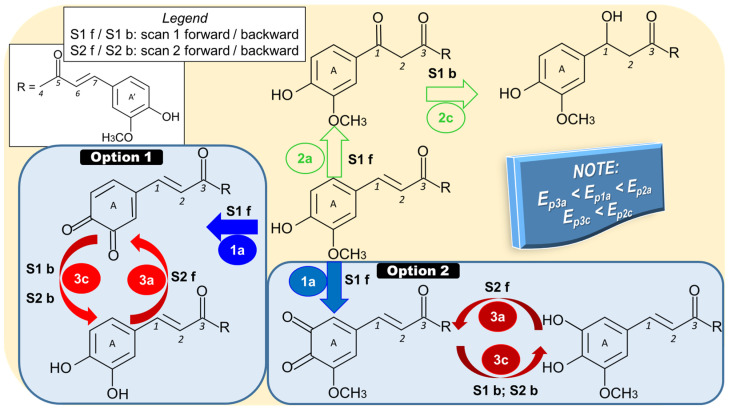
Possible CU electro-oxidation mechanisms.

If in the first voltammetric cycle, during the forward scan, the potential was swept in the anodic direction up to around 1.000 V or more, the cyclic voltammogram of CU usually showed an oxidation peak (1a) located at potentials in the range of 0.500 to 0.800 V, and only in a few cases a second signal (2a) occurred at more anodic potentials [95,99]. During the reverse scan, a cathodic peak (3c) was recorded between 0.300 and 0.500 V, for which, starting with the second voltammetric cycle, a paired anodic peak (3a) appeared in the same potential range, while peaks 1a and 2a disappeared. Peak 1a was attributed to the irreversible oxidation of CU with the formation of an o-quinone derivative (Figure 4) through an ECE mechanism, meaning that the phenolic hydroxyl was oxidized (electrochemical step) with the generation of a phenoxy radical, which underwent hydrolysis in the ortho-position resulting catechol (chemical step). The latter compound was immediately oxidized to o-quinone (electrochemical step) [95,100]. Although this is almost unanimously accepted, there are studies that revealed that this process involved 2e^−^ and 2H^+^ [8,19,46,101], with a total 4e^−^ and 4H^+^ per molecule [93,102,103], and others mentioned that the ratio between the number of protons and that of electrons participating in the electrode process was 1/2 [3,6,18,96,97,98,104,105]. On the other hand, some mechanisms indicated the oxidation of the 3-methoxy-4-hydroxyphenyl moiety to the corresponding o-benzoquinone substituent (Option 1 in Figure 4) [3,6,8,18,46,96,97,98,102,104,105], and, according to other reports, the methoxy group was not involved in the reaction generating peak 1a (Option 2 in Figure 4) [93,99,101,103]. These two different approaches are based on the fact that the initially formed phenoxy radical has two mesomeric forms, which subsequently led to the formation of the 3,4-dihydroxibenzene and 3,4-5-methoxy-dihydroxybenzene derivatives, respectively [100]. The absence of peak 2a in the voltammograms of capsaicin (which contains the guaiacol group but has no conjugated double bonds in the aliphatic side chain) led to the conclusion that it was generated by the oxidation at the double bond existing in the aliphatic hydrocarbon chain of CU and FA. This peak, which appeared at higher potentials, was due to the oxidation, after hydroxylation at positions 1 and/or 7, generating a product that participated in redox reactions corresponding to peaks 2a and 2c [95]. In another report, it was mentioned that CU oxidation signal 2a, from higher anodic potentials, could also be due to the oxidation of the enol group from the aliphatic chain linking the two aromatic ends of CU [94]. The disappearance of peaks 1a and 2a starting with the second potential cycle was a result of the electrode surface passivation by the CU oxidation products, so that this was no longer available for CU oxidation [19,95,98]. The adsorption of CU oxidation products at GCE was confirmed by the existence of peak 3a in the DPVs obtained for the blank solution at the washed GCE after several DPV recordings in the CU solution [95]. Regarding the pair of peaks 3a/3c, researchers unanimously attributed it to the reversible redox couple o-benzoquinone (product of CU oxidation in the process generating peak 1a)/catechol that involved the transfer of 2e^−^ and 2H^+^.

Moreover, some studies employed voltammetry [93], chronoamperometry [18,96], and chronocoulometry [19,97] to determine the CU diffusion coefficient, whose values varied from 9.35 × 10^−7^ to 4.05 × 10^−5^ cm^2^/s.

## 3. CU Electrochemical Analysis

### 3.1. Electrochemical Sensors and Methods for CU Quantification

Electrochemical methods are versatile tools for the rapid and sensitive quantitative determination of electroactive compounds, exploiting the direct proportionality between the peak current and the analyte concentration. Several voltammetric methods were reported in the literature for the quantification of CU based on its oxidation or reduction signals. Almost all of these methods used electrodes modified with carbon-based materials like CNTs, Gr, GO, metal- and metal-oxide-based NPs, polymers either as such or molecularly imprinted, or combinations of different modifiers in order to improve the sensitivity and selectivity of the determination. For example, the MnO_2_-c-MWCNTs/GCE obtained after drying the MnO_2_-c-MWCNTs suspension applied on the GCE surface presented a 2.5 times greater electroactive surface area as the bare GCE and more than 125 times lower R_ct_ value, which led to a shift of about 0.050 V in the negative direction of CU oxidation potential and to a more than 10 times higher peak current due to the synergistic electrocatalytic effect of MnO_2__NPs and c-MWCNTs [19]. Owing to a 3.71 times larger electroactive surface area and a faster electron transfer rate, 4 times enhanced peak currents were obtained by modifying the GCE surface with NSrGO/Ru@Au_NPs suspension and drying under an IR lamp [86]. At the Az-rGO@MWCNTs/GCE, prepared by dropping the Az-rGO@MWCNTs suspension at the electrode surface and air drying, CU redox peaks were 3-fold higher and shifted negatively with 0.021 V (anodic peak) and 0.083 V (cathodic peak) in comparison to the bare electrode [105]. A GCE was modified by drop casting with magnetic Fe_3_O_4__NPs covered by an MIP obtained by self-polymerization at room temperature of the biocompatible material Zein in the presence of CU acting as a template. Subsequently, CU was extracted from the polymeric matrix with methanol:acetic acid (9:1 *v*/*v*) solution in order to create the size- and shape-specific recognition sites, which enabled a more sensitive (a nearly 10-fold increase in the CU peak current in comparison to that recorded at unmodified GCE) and selective detection of the analyte [106]. A GCE modified also by drop casting with a CuCo_2_O_4_-*N*-CNTs and P-GO water–ethanol suspension was subsequently covered with an electrogenerated pCys film imprinted with CU. The template molecule was removed from the pCys matrix by CV in 0.10 mol/L KCl solution. The thus obtained pCys_MIP-CuCo_2_O_4_-*N*-CNTs-P-GO/GCE presented a low R_ct_ (4 Ω) compared to GCE (339 Ω) and a 4 times larger surface area, which, together with the high electron transfer ability of the nanocomposite and the cavities from the MIP, led to lower peak potentials separation and higher currents. The selectivity and sensitivity of this sensor towards CU were estimated by calculating the association constants of the polymeric binding sites, the obtained values being in the same range with those reported in the literature for other MIP-modified electrodes [76]. Compared to bare CPE, the electropolymerization of titan yellow at its surface led to a 7- and 12-fold enhancement of CU anodic and cathodic peaks, respectively [90], while the inclusion of a pMAA_MIP into the carbon paste generated a 4.5 increase in CU oxidation current [21]. The presence of CdO_NPs and of the ionic liquid 1,3-dipropylimidazolium bromide in the carbon paste matrix generated a CU anodic peak shifted negatively with 0.060 V and with the intensity more than triple compared to that occurring at CPE [96]. An interesting procedure was used to obtain PACO_MIP/GCE. First, the CU-containing polymer was prepared in DMF/H_2_O solution by bulk polymerization using PACO as monomer, CU as template, ethylene glycol dimethacrylate as cross-linker, and azobisisobutyronitrile as initiator. After 5 h of reaction, the solution was dropped at the GCE surface and heated at 65 °C for 10 h. Subsequently, this modified GCE was subjected to electropolymerization by CV in a solution containing N,N’-methylene bisacrylamide and ammonium persulfate, and then CU was removed from the polymer matrix by several extractions in acetic acid:methanol (1:1 *v*/*v*) solution. This sensor was employed for the indirect determination of CU by monitoring the decrease in the FCM oxidation peak with increasing CU concentrations, which presented a linear dependence [56]. These are only some examples of the ways of preparation and the generated sensing improvements in modified sensors for CU determination in comparison to bare electrodes. The performance characteristics of the electrochemical sensors for CU quantification reported in the literature in the last 20 years were summarized in Table 1.

### 3.2. CU Voltammetric Quantification in the Presence of Other Electroactive Species

Food samples are complex matrices that may contain, aside from CU, several other compounds, among them being vitamins, vanillin, and sometimes even not-allowed dyes, like metanil yellow. CU and vitamin B2 were determined simultaneously via DPV at pTY/CPE [90] and SDS/CNTsPE [18] due to the large separation between the oxidation peak potentials of the two analytes, namely of 0.666 V (E_pCU, pTy/CPE_ = 0.173 V and E_pVitamin B2, pTy/CPE_ = −0.493 V versus SCE) and about 0.540 V (E_pCU, SDS/CNTsPE_ = ~0.150 V and E_pVit B2, SDS/CNTsPE_ = −0.386 V versus SCE), respectively. The similar slopes of the regression equations describing the calibration plots of the SWV determination of CU at CdO-IL/CPE in the absence and in the presence of vitamin B9 as well as the 0.320 V difference between the peak potentials of the two analytes (E_pCU, CdO-IL/CPE_ = 0.420 V and E_pVitamin B9, CdO-IL/CPE_ = 0.740 V versus Ag/AgCl) enabled the analysis of these species one in the presence of the other [96]. CV and DPV simultaneous determination of CU and vanillin was reported to be possible at a pGA/CNTsPE, the peak potentials being separated by approximately 0.400 V [89]. There are often situations where CU used for foods is adulterated with metanil yellow, which is a harmful monoazo dye. Therefore, it is useful to have methods that allow differentiation between this compound and CU and its sensitive and fast detection in these complex samples, even if it is present in small amounts. DPV curves recorded at a CQDs/GCE for a mixture of CU and metanil yellow presented oxidation peaks characteristic for metanil yellow at 0.069 V and 0.208 V and for CU at 0.288 and 0.558 V versus SCE, respectively. In the presence of 1.00 × 10^−6^ mol/L CU, metanil yellow was determined in the concentration range 6.00 × 10^−8^–5.00 × 10^−5^ mol/L, while CU concentrations comprised between 4.00 × 10^−7^ mol/L and 1.00 × 10^−5^ mol/L were assessed at a fixed concentration of 1.00 × 10^−6^ mol/L metanil yellow [84].

An interesting voltammetric application was the determination of CU in various turmeric brands. This goal was achieved by exploring data analysis (radar plot, box plot, principal component analysis, linear discriminant analysis plot, and separability index) for the processing of the cyclic voltammetric results obtained at pTMS_MIP/CPE for five types of turmeric powder [111].

Cyclic voltammograms recorded at BDDE for CU and BDMCU in tetrabutylammonium hexafluorophosphate–acetonitrile solution presented no cathodic and two anodic signals (at 0.400 V and 1.900 V for CU and at 0.600 V and 2.000 V versus Ag/AgCl for BDMCU, respectively) whose peak currents varied linearly with the analytes’ concentrations. However, the method could not be applied for the simultaneous determination of the two electroactive species because, in the cyclic voltammogram of their synthetic mixture, the peaks were shifted and overlapped [114]. The DPV at HMDE quantitative determination of CU, DMCU, and BDMCU was possible by using the HPSAM. The LODs and the accuracy expressed as percentage relative error of the HPSAM-DPV method were 6.00 × 10^−7^ mol/L CU, 5.70 × 10^−7^ mol/L DMCU, 4.20 × 10^−7^ mol/L BDMCU, and less than 7.00%, respectively. The method was applied to the assessment of the three curcuminoids in two turmeric spices and a drug sample and the results were in good agreement with those obtained by HPLC [78]. Electrochemical detection at a GCE was applied for the simultaneous HPLC quantification of CU, DMCU, and BDMCU in *Curcuma longa* L. [115].

It was shown that piperine increases CU absorption efficiency in humans, and therefore these compounds can coexist in food and food supplements. A SWASV study at Ce-BDC-MOF-NPs/GPE emphasized that piperine was not oxidized in the anodic scan, but CU presented a signal at 0.520 V, while, in the cathodic scan, the mixture of the two species showed two peaks, at 0.520 V and −0.840 V versus Ag/AgCl, which were attributed to the reduction of CU and piperine, respectively. Thus, this method allowed the CU determination without any interference of piperine [91].

Doménech-Carbó et al. [116] discussed the possibility of examining curcuma and safflower dyes in archeological and artistic microsamples by solid-state SWV using PWIGE, on the surface of which a few micrograms of the dyes were immobilized in contact with aqueous supporting electrolyte (ABS or PBS). Based on the characteristic peaks, curcuma can be distinguished from safflower and from other flavonoid, indigoid, and anthraquinonic dyes.

### 3.3. Application of Voltammetric Methods in CU Release Kinetics Studies

The profiles for the in vitro release of CU from Zein electrospun fibers loaded with CU were established through monitoring the peak current of the CU CV anodic signal from 0.500 − 0.600 V versus Ag/AgCl, which was attributed to the reversible oxidation of the phenolic moiety, thus confirming the fact that CU maintained its antioxidant activity even after encapsulation in the Zein fibers [103]. The CU release from MBA_pAAM hydrogel was investigated by DPV measurements of the CU oxidation peak current during 10 h. The fact that the peak potential (0.900 V versus Ag/AgCl) remained unchanged suggested that the hydrogel prevented CU degradation [107].

## 4. Electrochemical Investigation of CU Biological Activities and Its Interactions with Various Chemical Species

### 4.1. Antioxidant Activity

Despite the fact that the AOC and the AOA, respectively, are determined by various methods, most of them being based on spectrometric measurements [117], these properties of chemical species are related to their ability to donate electrons, and therefore electrochemical methods like voltammetry (CV, DPV, SWV) and coulometry are useful tools in investigating them. Alam et al. [118] made a good and complete comparison between spectrometric and chromatographic methods on the one side and the electrochemical ones on the other side, applied in the assessment of the AOC of plant and fruit extracts. The authors emphasized sensitivity, simplicity, and rapidity, in both stationary and flow systems, as the main advantages of the electrochemical methods, but their reduced selectivity allows only the estimation of the total AOC of a sample. CV is the electrochemical technique most often applied to study the antioxidant characteristics of a sample. It is known that, the lower the anodic half-wave (CV)/peak (DPV) potential, the higher the AOA of the compound. The specific parameters of a voltammogram can be used to characterize a sample from the point of view of its antioxidant properties. According to Chevion et al. [119], the value of the half-wave potential of the anodic signal can be correlated with the reducing power and the radical scavenging capacity of the compound(s) and the peak current with the antioxidant(s) concentration, while the area under the anodic peak could be associated with the total AOC of a sample. Thus, based on the different reducing power of the various molecules, reflected in their oxidation (peak) potential, Blasco et al. [120] pointed out that electrochemical methods, i.e., amperometry, may have a certain degree of selectivity, and defined the “electrochemical index” according to which, by selecting the proper detection potential of 0.800, 0.500, and 0.300 V (versus Ag/AgCl, PBS pH 7.00), a distinction between the “Total natural antioxidant index”, “Intermediate antioxidant species”, and “High antioxidant compounds”, respectively, can be made. It was recently reported that DPV was able to discriminate between phenolics with high (polyphenolic acids like caffeic and gallic acid) and intermediate (naringin and naringenin bioflavonoids) AOA [121]. However, very often, the advantages of experimental methods (spectroscopic, chromatographic, electrochemical) and theoretical calculations are exploited together in order to establish the AOC of a sample.

The beneficial biological and pharmacological effects of CU are strongly correlated with its antioxidant and free-radical (ROS like superoxide anion, hydroxyl, and peroxyl radicals and NOS like nitric oxide and peroxynitrite species) scavenging properties, which further depend to a very high degree on its redox behavior. The reaction mechanisms (HAT, ET, and/or PT) and the sites (the two phenol rings and/or the CH_2_ group from the β-diketone moiety) involved in CU AOC are still under discussion and investigation using both theoretical and experimental approaches.

CV investigations of CU and o-methoxyphenol carried out at GCE in PBS pH 6.00 and pH 8.00, respectively, correlated with theoretical calculations of the bond-energy dissociation enthalpy, led to the conclusion that, in acidic medium only, for the phenolic hydroxyl group, while in alkaline environment, both the phenolic −OH and the –CH= from the β-keto-enol moiety are contributing to CU AOA. These results were also confirmed by monitoring the potential change of an oxidized polyaniline-modified electrode immersed in CU containing PBS pH 6.00 and pH 8.00, respectively, and by ESR measurements. The employed electrode was a GCE or a graphite fiber potentiostatically covered with polyaniline and subsequently treated by CV in order to ensure that polyaniline was in the oxidized form [122]. Based on CU tautomerization equilibrium, the CV peak potentials obtained for CU at GCE in acetonitrile (0.800 V versus Ag/AgCl) and in NaOH (0.350 V versus Ag/AgCl) solutions were attributed to the irreversible oxidation of the phenolic OH sites in the keto and enol CU isoform, respectively. Experimental and quantum computational results indicated that, in physiological conditions, where both CU tautomeric forms coexist, two phenolic hydroxyl groups and both HAT and ET mechanisms are involved in its AOA [123].

To shed more light on the mechanism on which CU AOA relies, Jha et al. [88] synthesized CU structurally modified analogues and compared their CV behavior at a polycrystalline gold electrode with that of CU. In order to show the involvement of the −CH_2_− group in CU redox characteristics, changes were performed at the aliphatic link; namely, the β-diketo and the −CH_2_− groups were modified by synthesis of some substituted pyrazole containing derivatives and of Knoevenagel condensate of CU, respectively. The importance of the phenolic –OH group to CU AOA was investigated by CV studies performed on half-CU and dimethyl-CU. The results pointed out that both the central methylene and the phenolic hydroxyl groups contribute significantly to CU AOA. Another study reported the exploitation of the CV and MS measurements, as well as DFT calculations to assess the reactivity of mono- or di-substituted CU derivatives, obtained by esterification with first- and second-generation polyester dendrons and glutaric anhydride, towards the electrogenerated superoxide anion. All tested compounds presented AOC, demonstrated by their reaction with O_2_^▪−^, which followed a PT mechanism excepting the disubstituted OH-terminated second-generation derivative, for which the mechanism was based on HAT reactions [124].

A commonly used method to appreciate the AOC is the FRAP assay, which consists of the reduction of Fe^3+^ to Fe^2+^ by the antioxidant. Most commonly, the reaction is monitored spectrophotometrically by measuring the absorbance at 593 nm of the resulted blue Fe^2+^–tripyridyltriazine complex [125]. Ziyatidinova et al. [126] reported the coulometric evaluation of FRAP of spices. In this procedure, Fe(CN)_6_^3−^ electrogenerated at constant current in alkaline medium oxidized phenolic antioxidants from spices. Micellar Triton X 100 solutions of each individual phenolic compound were coulometrically titrated with the electrogenerated Fe(CN)_6_^3−^ ions, and the stoichiometric coefficients of the reactions were determined. The quantity of electricity involved in the titration represented the FRAP of the sample. All tested species, excepting CU, reacted rapidly and quantitatively. This behavior of CU, explained by its high hydrophobicity and low solubility in the micellar medium, did not allow the assessment of its reactivity towards Fe(CN)_6_^3−^ in these conditions. Consequently, applying this method, the micellar Triton X turmeric extract, which had a high content of CU, presented the lowest FRAP value among the 16 tested spices. In another investigation, CU radical scavenging ability was tested by voltammetric, coulometric, and spectrometric measurements using the superoxide anion radical electrogenerated in acetonitrile from the dissolved oxygen. The results pointed out that each mol of CU can react with 6 moles of O_2_^▪−^ and the mechanisms of the free radicals’ elimination in the presence of CU and of the enzyme superoxide dismutase are very similar [127].

The electrochemical behavior of 15 individual polyphenolic antioxidants, among them being CU, was investigated in the same conditions (CeO_2__NPs-Brij^®^ 35/GCE in PBS pH 7.40 in the presence of Brij^®^ 35) as those employed for the DPV analysis of 20 spice extracts in micellar medium of the Brij^®^ 35 surfactant, with the aim to use their anodic peak potentials to assign the oxidation signals that occurred in the voltammogram of each tested spice. The AOCs of the spice extracts were assessed by considering the total area of the DPV oxidation peaks recorded for each spice. The highest AOC, expressed as mg of gallic acid/g dry spice, was found for cloves (153.0 ± 5.0), followed by black pepper (26.0 ± 2.0), while that of turmeric (20.0 ± 1.0) and black curcuma (3.8 ± 0.1) was moderate and low, respectively [128]. The AOCs of aqueous and ethanolic extracts of leaves and rhizomes of plants from the *Zingiberaceae* family, including *Curcuma longa*, were determined electrochemically by CV and spectrophotometrically by the DPPH assays. Despite the low positive correlation (r = 0.22) between the results of the two methods, both indicated that the leaves of the tested plants present antioxidant activity [129]. It was demonstrated by the DPPH assay that nanofibers of poly(lactide–glycolide)/chitosan loaded with CU exhibited AOC that increased with higher CU contents due to the existence in its molecule of the phenolic hydroxyl and the methylene groups. CV measurements emphasized the presence of CU-concentration-dependent peaks corresponding to the quinone/hydroquinone redox couple, the anodic one indicating that CU AOC was not altered through encapsulation in the fiber matrix. This aspect was important for the potential use of these nanofibers as CU delivery systems with therapeutic action [130].

### 4.2. Antitumoral Activity

**Figure 5:**
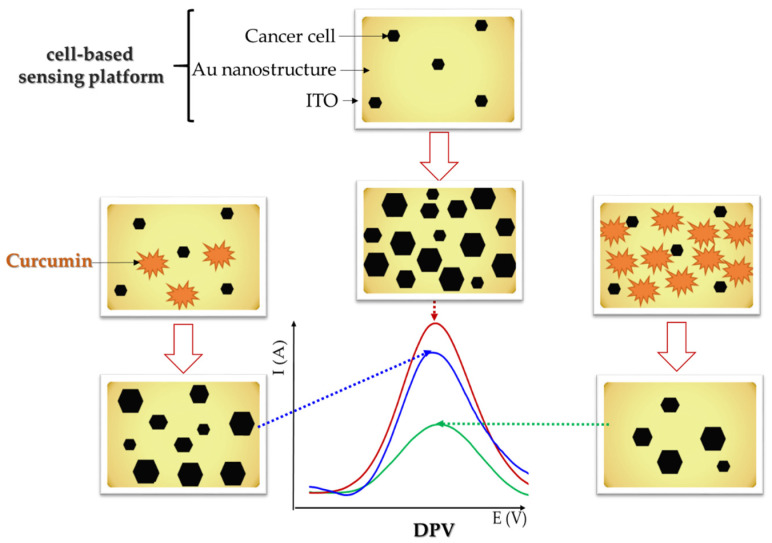
Schematic representation of DPV assessment of CU antitumoral effects.

Cancer cells contain redox proteins that allow the use of electrochemical methods to quantitatively determine their viability based on their redox signaling responses [22]. Cancer cell growth analysis using electrochemical impedance measurements at Au microelectrodes modified with reduced graphene oxide and polyaniline emphasized the cytotoxic effects of CU on human gastric cancer cells (MKN-1) [131]. On the other hand, rapid DPV detection (less than 3 min) at cell-based sensing platforms fabricated on gold-modified ITO substrate was employed to evaluate CU anticancer properties on a multicellular brain tumor model [132] toward liver cancer cells (HepG2) [133] and human glioblastoma (U87MG) [22]. The principle of the DPV assessment of CU antitumoral activity is based on monitoring the peak current of the cancer cell culture platform in the absence and presence of CU (Figure 5), considering that the peak intensity is proportional to the number of cancer cells. The decrease in the DPV peak current in the presence of increasing CU concentrations was correlated with decreased cell viability, indicating the cytotoxic effect of CU to cancer cells. At concentrations higher than 3.00 × 10^−5^ mol/L, CU was toxic to U87MG cells and the DPV signal decreased with increasing CU concentrations up to 1.00 × 10^−4^ mol/L [22].

### 4.3. Antibacterial Activity

Pyocyanin is a virulence factor secreted by *Pseudomonas aeruginosa.* The inhibiting effect of CU on the formation of the *Pseudomonas aeruginosa* biofilm was demonstrated by the decrease in the pyocyanin DPV signal recorded in the presence of CU at a gold-nanoparticles-modified microelectrode incorporated in a biosensor chip [134].

### 4.4. CU Interaction with Metal Ions

CU chelating properties are due to the existence of the β-diketo/keto-enol moiety in its molecule. Thus, CU ability to form complexes with several metal ions has been exploited for different purposes; e.g., the nano Zn^2+^–CU complex was recently applied for the efficient in situ removal of certain bacteria from water samples [135]. On the other hand, it is known that some metals, acting as micronutrients, are necessary for the living organisms in low concentrations, at high concentrations becoming toxic. Studies have demonstrated that CU ability to form complexes with metal ions, like Cu^2+^, can reduce or even prevent this negative effect [136]. CU was determined by AdSCV from a mixture containing other guaiacol derivatives, like capsaicin, after its separation by precipitation with NiCl_2_ in alkaline medium. For the AdSCV analysis, CU was subsequently released from the Ni:CU complex by treatment with an acidified ethanolic solution [110]. A Ni^2+^–CU complex was electropolymerized on the GCE surface and characterized by CV. The growth of the conductive polymeric film was observed by the increase in the peak currents corresponding to the Ni^2+^/Ni^3+^ redox pair. It is worth mentioning that Ni^2+^ oxidation to Ni^3+^ took place only in the poly(Ni^2+^-CU) film, not in the monomer. The GCE modified with poly(Ni^2+^-CU) film was used to investigate the electrocatalytic properties of the polymer towards oxidation of aliphatic alcohols [137], amino-acids [138], glucose [139], fructose [140], and non-steroidal anti-inflammatory drugs [141], while a CPE modified with Ni^2+^–CU complex obtained similarly, by electropolymerization, was applied for amoxicillin quantification [142].

A Au^3+^-CU film was galvanostatically electrodeposited at GCE acting as cathode, while a Au wire served as reactive anode. The metal from the electrode was oxidized to Au^3+^, which, in the presence of the Cl^−^ ions from the HCl used as supporting electrolyte, formed the chloroaureate salt, which subsequently reacted with CU to generate the Au^3+^–CU complex. At the cathode, Au^3+^ was reduced to Au^0^, resulting in the Au–CU nanocomposite with high electrocatalytic activity and low Arrhenius energy towards ethanol and methanol electro-oxidation in basic environment [143].

Electrochemical methods were also applied, sometimes together with the spectrometric ones, to investigate the formation of complexes between CU and metal ions and/or to characterize them.

In acidic media (pH 3.00), the changes in the shapes and potentials of peaks observed in the CV recordings of mixtures of CU and Fe^2+^ and CU and Fe^3+^, respectively, in comparison with the cyclic voltammograms obtained in the same conditions for each individual component (CU, Fe^2+^, and Fe^3+^) indicated (i) the possible formation of a complex between CU and Fe^3+^; (ii) the existence of some chemical interactions between CU and Fe^2+^; and (iii) the fact that CU adsorption at the electrode surface was influenced by the presence of the two ions. Similarly, CV studies performed at pH 10.00 showed that, in alkaline media, the presence of the investigated ions had no influence on CU adsorption at the CPE surface and Fe^3+^ was reduced by CU, thus confirming the results obtained by spectrophotometry, according to which the two systems, CU and Fe^2+^ and CU and Fe^3+^, have similar behavior [144]. The electrochemical behavior of the Fe^3+^–CU complex and its reactivity upon the superoxide, tested in the presence of this radical, investigated at Pt electrode in DMSO, revealed that this complex has the ability to eliminate free radicals [145]. Similar conclusions were drawn for the Fe^3+^–CU–oxime complex. The authors suggested that CU and CU–oxime may be used to treat iron overload [146].

Comparative CV studies at bare CPEs and modified with CU, SASPM_NPs, and CU-modified SASPM_NPs, respectively, emphasized that CU presence led to an enhanced electroactive surface area. Moreover, the reversibility degree of CU redox behavior was higher and very stable even after 100 voltammetric cycles or after applying a constant anodic potential for 3 h, when it was bonded to the Fe_2_O_3_ from the SASPM_NPs, due to its good chelating properties. The CU_ SASPM_NPs/CPE responded linearly to H_2_O_2_ and NADH concentrations. The sensitivity of the CU_SASPM_NPs/CPE compared to that of the electrode without CU was higher towards H_2_O_2_ and lower with respect to NADH. This observation was explained by the CU electron donating capacity, which had a higher effect on H_2_O_2_ reduction than on NADH oxidation. The authors considered that CU_ SASPM_NPs could be developed as an alternative CU-delivery system [147].

The stability constant (1.58 × 10^−10^ L/mol) and the stoichiometric ratio (1:3) of the Cr^3+^–CU complex were determined by DPV using the difference between the reduction peak potential of Cr^3+^ in the absence and in the presence of CU [148].

Cyclic voltammograms at HMDE emphasized for the Al^3+^–CU complex three irreversible reduction peaks generated by adsorption-controlled processes [149]. The presence of Cu^2+^ ions affected the cathodic peaks recorded for CU by DPV at HMDE; i.e., the signal observed at −1.100 V versus Ag/AgCl decreased and that from −1.600 V versus Ag/AgCl was suppressed. At the CPE, the presence of Cu^2+^ led to a smaller CU characteristic reduction peak at 0.300 V versus Ag/AgCl and to the appearance of a new peak at 0.000 V versus Ag/AgCl. These observations indicated that there were interactions between Cu^2+^ and CU with the possible formation of a complex between the two species [11]. The cyclic voltammograms recorded at GCE for Cu^2+^ ions in the presence of CU in 0.10 mol/L KCl solution presented shifted peaks and supplementary peaks of a redox couple (E_pa_ = 0.390 V; E_pc_ = 0.320 V versus Ag/AgCl) attributed to CU, suggesting the formation of a Cu^2+^–CU complex. The increase in the peak currents with repetitive cycling indicated the formation of a conductive polymer at the GCE surface [150]. In another study, the complexation between Cu^2+^ ions and CU was demonstrated by the decrease in the intensities of the quinone/hydroquinone peaks of CU immobilized at a SWCNTs/GCE after incubation with Cu^2+^ ions. The formation of a Cu^2+^–CU complex was also confirmed by the presence of a new couple of redox peaks in the cyclic voltammogram of Cu^2+^–CU–SWCNTs/GCE in comparison to that of the CU–SWCNTs/GCE [151].

Conductometric analysis of CU complexes with Mn^2+^, Zn^2+^, Fe^3+^, and Cr^3+^ showed that they are non-electrolytic, while CV measurements emphasized the redox potentials of the complexes depended on CU electron donating properties [152].

### 4.5. CU Interaction with DNA

CU interaction with ct-dsDNA either in solution or immobilized at a pre-treated (1 min at 1.700 V) CPE was investigated in ABS pH 5.00 by monitoring the specific guanine oxidation peak via transfer DPAdSV and the DNA tensametric signals by ACV at HMDE as a complementary technique. The guanine signal of ct-dsDNA decreased in the presence of CU, the decrease being more significant with increasing concentrations of the polyphenol, indicating that there was an interaction between the two compounds. The authors explained the strong CU-ct-dsDNA interaction by the fact that, in mild acidic media, the guanine-protonated cytosine pair eliminated an amino group from the minor groove and thus allowed sterically the interaction with CU. The decrease in the guanine signal was lower in the case of ct-dsDNA immobilized onto the CPE compared to the situation in which the interaction between the two compounds took place in solution, most probably due to the fact that the helical part attached to the electrode surface was less accessible for the interaction with CU. At the HMDE, ct-dsDNA presented two structure-sensitive tensametric signals, E_pI_ = −1.180 V, E_pII_ = −1.420 V versus Ag/AgCl. Peak I was due to the reorientation of the ct-dsDNA segments adsorbed at the electrode surface, while peak II was sensitive to the conformational changes of the ct-dsDNA double helix. When the interaction between CU and ct-dsDNA was realized in solution, the decrease in these peaks was smaller, as in the case of ct-dsDNA adsorbed at the HMDE, where the interaction was hindered sterically [153].

CU DPV oxidation peak was exploited to study the hybridization of oligonucleotides (probe) containing only one of the base types (poly A, poly T, poly C, and poly G), which were potentiostatically immobilized (5 min at 0.500 V in 1.00 × 10^−6^ mol/L probe in 0.01 mol/L Tris-buffer solution pH 7.00 containing 0.02 mol/L NaCl) onto the surface of an electroactivated PGE (5 min at 0.500 V in ABS pH 4.80), resulting in a biosensor denoted as probe–PGE. The interaction of CU with each of the oligonucleotides was investigated by measuring CU oxidation peak intensity before and after the hybridization with the target. The hybridization was performed by maintaining the probe–PGE for 5 min at 0.500 V in 1.00 × 10^−6^ mol/L complementary oligonucleotide (target) in 0.01 mol/L Tris-buffer solution pH 7.00 containing 0.02 mol/L NaCl, thus resulting in the target–probe–PGE. CU was accumulated keeping the corresponding electrode for 5 min in 1.80 × 10^−5^ mol/L CU in ABS pH 4.80 containing 0.10 mol/L NaCl. The CU DPV oxidation signal was higher at target–probe–PGE because more CU was accumulated at dsDNA by intercalation or by groove binding within the DNA double helix. Starting from the observation that CU interacted stronger with the hybridized oligonucleotides, the hybridization degree was assessed based on the difference between the oxidation peak current of CU accumulated at probe–PGE and at target–probe–PGE, respectively. The applicability of this biosensor was tested using short sequences of hIL-2 gene (target) as model. The CU peak current at the chIL-2-PGE increased with the concentrations of hIL-2, two linear ranges being obtained (5.00 × 10^−11^–1.00 × 10^−9^ and 1.00 × 10^−8^–1.00 × 10^−6^ mol/L) and an LOD of 1.20 × 10^−11^ mol/L hIL-2 [154].

**Figure 6:**
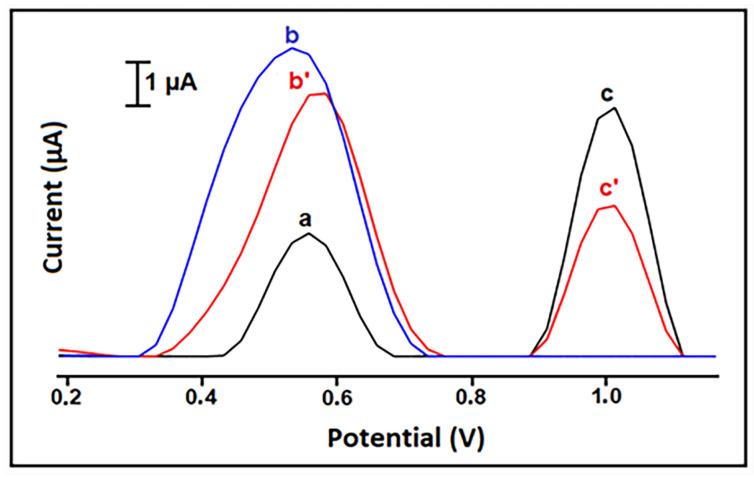
Voltammograms representing the oxidation signal of 10 µg/mL CU and the oxidation signal (i.e., guanine signal) of 25 µg/mL ct-dsDNA measured before and after 3 min interaction: (a) the control signal measured by HaP-IL-PGE, oxidation signal of CU (b) before, (b’) after interaction, oxidation signal of guanine (c) before, (c’) after interaction [109].

CU interaction with DNA was assessed using disposable HaP-IL/PGEs on which ct-dsDNA was immobilized. The thus obtained DNA-based biosensor was introduced into the CU solution for interaction. The changes in CU and guanine oxidation signals were monitored via DPV (Figure 6). The same procedure was applied using also PCR samples and the results were in good agreement with those obtained by gel electrophoresis [109].

It was shown that, in the presence of Cu^2+^ ions, CU damaged the DNA molecule due to the formation of a Cu^2+^–CU complex that interacted with DNA and the generation of reactive hydroxyl radicals. The interaction of the Cu^2+^–CU complex with ct-dsDNA was investigated via DPAdSV at CPE, and it was confirmed by the increase in the CU anodic signal at 0.600 V versus Ag/AgCl and the considerable drop in the CU (0.300 V versus Ag/AgCl) and guanine (1.200 V versus Ag/AgCl) oxidation peaks [11]. Cyclic and differential pulse voltammograms of two mononuclear complexes of Cu^2+^ with CU, namely CuCU and CuCU_2_, showed that the addition of DNA to the complexes generated a positive shift in the formal potentials, indicating that the complexes were strongly bonded in the DNA groove and a significant decrease in the peak currents due to the slow diffusion of the large complex DNA molecule in comparison to the smaller CuCU and CuCU_2_ structures [155]. In the case of a Cu^2+^ complex with CU and 4,7-diphenyl-1,10-phenanthroline, CV and DPV studies at CPE revealed that the complex intercalated between the DNA strands, the binding constant being 3.50 × 10^4^ [156].

CV investigations at HMDE in Tris-HCl buffer pH 7.20 pointed out that, in the presence of ct-dsDNA, the reduction peaks of the Al^3+^–CU complex decreased and shifted towards more cathodic potentials due to the interaction of the complex with the DNA, making the reduction process more difficult. Moreover, CV results allowed the calculation of the intrinsic binding constant of 2.60 × 10^4^ and the binding site size of 2 for the Al^3+^-CU interaction with ct-dsDNA [149].

### 4.6. CU Interaction with Other Molecules

Martínez-Guerra et al. [77] researched CU interaction with β-CD employing both CV and spectrophotometry. They showed that, in the absence of β-CD, CU cyclic voltammograms presented two anodic peaks (at ~0.600 V and ~0.800 V versus Ag/AgCl), while, in the presence of the macrocyclic molecule, only one sharp and somewhat higher anodic signal (~0.800 V versus Ag/AgCl) occurred. The observation that, after 10 min, in the absence of β-CD, the first CU oxidation peak disappeared and the second one decreased drastically, while the signal attained in the presence of β-CD was only slightly diminished, indicated that β-CD had a stabilizing effect on CU through the formation of an inclusion complex. Correlating the results obtained from the first two voltammetric cycles recorded for CU in solution without and with β-CD, with the electrochemical and theoretical data previously published in the literature, the authors discussed in detail CU electrochemical behavior and proposed reaction mechanisms for the two situations.

## 5. CU Applications in the Development of Electrochemical Sensors

In addition to its well-known biological activities, CU also possesses optical and fluorescence properties as well as complexation and electrocatalytic abilities, making it useful in chemosensors development. A review published in 2019 discussed CU various sensing applications, with emphasis on luminescence-based detection modes [157]. A more recent paper showed CU utility in the assessment of environmental pollutants [158]. CU-based electrochemical sensors reported in the literature in the last 20 years will be briefly presented below.

CU exhibited electrocatalytic activity, and therefore it was used, either alone or in combination with other (nano)materials, to modify the electroactive surface of different electrodes in order to obtain sensors for sensitive detection of various chemical species.

CU was electrodeposited at the surface of electrochemically pretreated GCE bare or modified with MWCNTs, respectively. The GCE was electroactivated potentiodinamically in 0.100 mol/L NaHCO_3_ solution by cycling the potential 20 times in the range −1.00 to 0.550 V versus SCE. CU was electrodeposited from a 5.00 × 10^−5^ mol/L CU in PBS pH 8.00 by applying 16 potential cycles between −0.150 and 0.550 V versus SCE, with a scan rate of 0.100 V/s. The studies revealed that CU was not deposited at untreated bare GCE because of its smooth surface, while the high roughness of the electroactivated GCE surface resulted in fast electron transfer processes, which enabled CU electropolymerization, the formed polymeric film being bonded to the electrode surface by the functional active groups generated during the GCE electroactivation process. During the CU polymerization process, the anodic peak (E_pa_ = 0.400 V versus SCE) observed in the direct scan of the first cycle, attributed to the CU irreversible oxidation to the o-quinone derivative, decreased with increasing scan number till the complete disappearance after the sixteenth cycle. The signals assigned to the pair of peaks (E_pa,I_ = 0.140 V and E_pc,I_ = 0.120 V versus SCE) corresponding to the quinone/hydroquine redox couple increased, indicating the formation of the polymer at the electrode surface. The CU/GCE presented a high electroactive surface area and electrocatalytic activity towards the oxidation of p-acetaminophen and epinephrine, which enabled either individually or simultaneously selective quantification [159]. Hydrazine electro-oxidation at a CU-MWCNTs/GCE took place at a lower potential and the anodic signal was higher in comparison to those recorded at MWCNTs/GCE and bare electroactivated GCE, thus demonstrating the CU electrocatalytic activity [160]. A PGE was covered with a film of polyCU obtained by CV in PBS pH 7.40 [161] or in acetonitrile. Also, in non-aqueous media, in the first anodic scan, a broad ill-defined signal was observed at about 0.850 V versus Ag/AgCl, whose intensity decreased gradually in the subsequent scans. In the next cycles, the peaks of a redox couple (E_pa_ = 0.065 V and E_pc_ = −0.130 V versus Ag/AgCl) were observed. The enhancement of their peak currents with the scan number confirmed the formation of the CU polymeric film. The authors also proposed a mechanism for CU electropolymerization. The polyCU-modified PGE presented a higher electrocatalytic response towards hydroxylamine in comparison to the bare PGE [162]. Nayak et al. [163] discussed the cyclic voltammetric CU accumulation onto GCE bare and modified with various carbon-based materials (CNTs, oCNTs, graphite, and GO) and presented the mechanism for CU electrodeposition and the analytical performances of a polyCU-oCNTs/GCE for the simultaneous sensing of dopamine and serotonin. Dinesh et al. [164] reported the electrodeposition of a CU-quinone derivative at a CB/GCE by in situ electro-oxidation via CV in KCl-HCl pH 2.00 solution. LC–MS and EQCM analysis confirmed the presence of the CU-quinone derivative at the electrode surface. The thus prepared electrode exhibited electrocatalytic activity towards sulfide oxidation.

In another procedure, CU was immobilized at a MWCNTs-modified PWIGE by drop casting CU ethanolic solution at the electrode surface followed by its electropolymerization in PBS pH 7.00. CU cyclic voltammetric behavior during electropolymerization was similar to that described previously. Due to the synergistic catalytic activities of the polyphenol and the carbon nanomaterial, the obtained polyCU-MWCNTs/PWIGE enabled the sensitive determination of butylated hydroxyanisole [165]. Drop coating of the NiS_2_-rGo/SPCE with aqueous solution of CU_NPs was employed for the preparation of the CU_NPs-NiS_2_-rGO/SPCE applied to the simultaneous quantification of methyl parathion and 4-nitrophenol [166]. CU-Ag_NPs-SDS-MWCNTs/GCE was also obtained by coating the electrode surface with CU-Ag_NPs-SDS-MWCNTs suspension and subsequent drying in ambient conditions [167].

Another category of electrodes modified with CU includes the ones where the modifier was a CU complex with metal ions. Most of these sensors were obtained by the electropolymerization of the metal ion–CU complex onto the electrode surface [30,137,138,139,140,141,142], but there were also other reported methods applied to change the sensor surface. For example, a Ni(CU)_2_ complex was obtained by chemical synthesis and the modified GCE was prepared by drop casting a Ni(CU)_2_/GO suspension on its surface and air drying [168].

CU was also employed to stabilize Ag-NPs used in the preparation of a SPCE modified with CU−Ag_NPs-coated reduced graphene oxide magnetic spinel (FeCo_2_O_4_) nanosheets for the simultaneous determination of p-nitrophenol and hydrazine [169].

It is worth mentioning that, for the polyCU-MnO_2_-Gr-modified GCE, CU played the role of both electrochemical transducer and ion receptor, the platform being employed for the concomitant DPV analysis of Hg^2+^, CN^−^, and F^−^, based on the fact that the enol form of CU is able to detect anions via hydrogen bond, while the keto structure is responsible for the sensing of metal ions. It was observed that the addition of anions to the CU solution resulted in a decrease in CU oxidation peak and the appearance of a new signal at more cathodic potentials, assigned to the formation of the CU–anion complex. A similar behavior was observed in the presence of Hg^2+^ ions, with the only difference that the new anodic peak, attributed to the Hg^2+^–CU complex, appeared at more anodic potentials with respect to the CU oxidation signal. The peaks of the CU complexes increased linearly with the concentration of the added analyte [170].

To increase CU conductivity and its adherence to the electrode surface, as well as its bioavailability, CU was loaded into the zirconium-based MOF UiO-66. A CU-UiO-66/GCE with enhanced sensitivity towards methyl parathion was prepared by drop casting a CU-UiO-66 and Nafion methanolic solution on the GCE surface [171]. An immunosensor prepared by AFB_1_-BSA conjugate immobilized at a Au surface was developed for AFB_1_ detection using IgG immunoglobulin labeled with the composite CU@ZIF-8-PDA as a signal probe. CU was chosen due to its electrochemical activity, ZIF-8 was used to encapsulate CU and due to its large specific surface area and its strong adsorption properties, while the biocompatible PDA had the role to improve the conductivity and the sensitivity of the probe. The working principle of the sensor consisted of monitoring the DPV oxidation peak current of the CU@ZIF-8-PDA-labeled IgG bound to the anti-AFB_1_ antibodies linked to the AFB_1_-BSA immobilized at the Au electrode surface. Due to the competition between free AFB_1_ and AFB_1_-BSA for a constant amount of anti-AFB_1_ antibodies and the preference of the antibodies for the free AFB_1_, the amount of remaining antibodies that can bind to the AFB_1_-ABS immobilized at the Au electrode and, subsequently to the probe, was lower for higher concentrations of free AFB_1_. Therefore, the DPV signal decreased linearly with increasing concentrations of free AFB1 in the range 0.5 pg/mL to 10 ng/mL. This electrochemical immunosensor exhibited an LOD of 0.11 pg/mL and was applied for the AFB1 analysis in spiked rice and wheat [172].

An interesting paper-based analysis device containing molecularly imprinted polyacrylamide-coated CU_NPs was developed for dual electrochemical and fluorescence sensing of bisphenol A [173]. A device constituted of an ITO substrate modified with GQDs covered with electropolymerized CU was used as dual detection mode platform for the assessment of APO *e4* DNA, a biomarker for Alzheimer’s and several artery disorders. The amperometric quantification of APO*e4* DNA was possible due to the linear decrease in CU oxidation current in the presence of increasing analyte concentrations [174].

CU was used as an electrochemical indicator in an aptasensor based on thiolated aptamer immobilized at Au_NPs-FMWCNTs-IL-Chit/SPE for epirubicin detection. The assay principle was based on the following: (i) epirubicin and the complementary strand of the aptamer compete for the binding to the aptamer, epirubicin having a higher tendency in this respect, and (ii) CU binds to the grooves of dsDNA and its redox signal can be monitored by DPV. The CU redox signal decreased linearly with increasing epirubicin concentration in the range 7.00 × 10^−9^–7.00 × 10^−6^ mol/L. This can be explained by the fact that, if more epirubicin molecules interact with the aptamer, there remain fewer sites for the interaction with the complementary DNA, and thus the number of dsDNA and implicitly of CU molecules from the electrode surface decreased [175].

A SPE modified with CU nanoparticles functionalized with the trastuzumab antibody (Anti-CU_NPs/SPE) was developed as a disposable impedimetric cytosensor for the fast and sensitive quantification of breast cancer cells (BT-474). Trastuzumab is a monoclonal antibody for HER-2, an antigen found on the cancer cell membranes, which is overexpressed in breast cancer. CU was used as a surface modification agent that enables good binding of the antibody and its nanostructure, offering a large specific area with good adherence for the cells without altering the bioactivity. The interaction between the BT-474 cells and the Anti-CU-NPs/SPE was monitored by EIS. The detection principle was based on the fact that, when trastuzumab has bound to the HER-2 receptor, the R_ct_ value increased. There was a linear correlation between ΔR_ct_ and the cells’ concentration in the range 1.00 × 10^2^–8.00 × 10^4^ cells/mL, where ΔR_ct_ was the difference between the R_ct_ before and after the cell immobilization. The low detection limit of 65 cells/mL of the method suggested that it may be adequate for the early diagnosis of breast cancer [176]. The CV and EIS linear responses of a PC3/GCE to concentrations of NH_3_ (only EIS), methylamine, dimethylamine, and trimethylamine varying from 0.10 to 1.00 µg/g suggested the possibility to apply this sensor for the assessment of total volatile basic nitrogen content [177]. The performance characteristics and applications of other CU-based electrochemical sensors were summarized in Table 2.

## 6. Conclusions

This review not only summarized the data related to CU electrochemistry compiled from over 180 scientific papers published in the last 20 years but also discussed and compared them. The huge interest in this topic results from both CU importance in daily life and the inherent scientific, practical, and economical characteristics of the electrochemical sensors and techniques.

When it comes to an analytical method, one first thinks about its ability to detect and determine as accurately as possible one or more chemical species; electrochemical methods accomplish this well. From the detailed search of the literature, it emerged that, in the last 20 years, more than 40 papers presented new developed electrochemical sensors (most of them being modified electrodes) and methods for CU-sensitive quantification from turmeric rhizome, food, and biological samples, with LODs mainly below 1.00 × 10^−6^ mol/L, some reaching the picomolar level or even 2.00 × 10^−13^ mol/L CU.

Voltammetric methods also offered the possibility to investigate the CU interaction with chemical species like metal ions, radicals, DNA, and other molecules of biological significance, thus providing valuable information that allowed the understanding of some mechanisms underlying CU beneficial health effects (e.g., antioxidant, antitumoral). Thus, based on the existing literature data, each of these aspects were analyzed and comparatively discussed in dedicated sections.

Last but not least, the symmetrical chemical structure with important functional groups (guaiacol, β-diketo/keto-enol, conjugated double bonds) confers CU electrochemical and optical properties that have made this molecule useable in the development of chemical sensors. A special section of this review is addressed to the preparation and performance characteristics of CU-based electrochemical sensors developed for the detection of different chemical species.

All the information gathered in this up-to-date comprehensive review may inspire researchers in the field to continue their work towards designing electrochemical sensors with enhanced sensitivity and selectivity to enable CU determination in the presence of structurally related compounds. The development of disposable, eco-friendly, and cost-effective electrodes for the rapid and accurate routine analysis of CU in finite food samples or during the fabrication process may be of interest.

## Figures and Tables

**Table 1:** Experimental conditions and analytical performances of electrochemical methods reported in the literature for CU determination.

Technique	Electrode	Peak, E_p_ (V); Conditions	Linear Range (mol/L)	LOD (mol/L)	Sample	Ref.
		Reduction				
DPAdCSV	HMDE	−1.100 BRB pH 9.50 E_acc_ = −0.300 V; t_acc_ = 50 s	5.00 × 10^−9^–2.80 × 10^−7^	1.50 × 10^−9^	Human serum, turmeric	[83]
DPAdSV DPV	CPE HMDE	0.300 ABS pH 3.50 + 0.02 mol/L NaCl E_acc_ = 0.300 V; t_acc_ = 120 s −1.100 PBS pH 8.50 + 0.10 mol/L NaCl E_acc_ = −0.800 V; t_acc_ = 60 s −1.600 PBS pH 8.50 + 0.10 mol/L NaCl	5.76 × 10^−8^–2.74 × 10^−6^ 4.95 × 10^−7^–2.76 × 10^−5^ 9.60 × 10^−7^–4.84 × 10^−5^			[20]
		Oxidation				
CV	GCE	0.740 0.10 mol/L LiClO_4_ in ethanol	9.90 × 10^−6^–1.07 × 10^−4^	4.10 × 10^−6^	Spices	[94]
DPV	GCE	0.090 PBS pH 7.40 + 0.10 mol/L KCl	1.00 × 10^−7^–3.50 × 10^−6^	1.00 × 10^−7^	CU release from MBA_pAAM hydrogel	[107]
DPV	NiCl_2_/GCE	~0.350 PBS pH 4.00	1.00 × 10^−5^–6.00 × 10^−4^	1.09 × 10^−7^	Human blood serum	[85]
CV DPV	CQDs/GCE	0.288 PBS pH 4.50	8.00 × 10^−7^–1.00 × 10^−4^ 4.00 × 10^−7^–2.00 × 10^−4^	1.00 × 10^−7^	Turmeric powder	[84]
SWV	MWCNTs /GCE	~0.700 PBS pH 2.50 + CTAB	1.09 × 10^−6^–5.43 × 10^−6^	8.97 × 10^−7^	Curcuma longa	[14]
SWV	MWCNTs /GCE	~0.550 0.10 mol/L HCl t_acc_ = 10 min	1.00 × 10^−8^–5.00 × 10^−3^	5.00 × 10^−9^	Turmeric extract	[102]
SWV FFTSWV	MWCNTs /GCE Dy_NWs /CPE	~0.450 PBS pH 4.00 E_acc_ = −0.700 V; t_acc,SWV_ = 110 s; t_acc,FFTSWV_ = 0.4 s	1.00 × 10^−8^–1.00 × 10^−6^ 2.00 × 10^−9^–1.00 × 10^−6^	5.00 × 10^−9^ 5.00 × 10^−10^	Milk	[17]
AdSCV	MWCNTs /BPPGE	0.740 BRB pH 1.81 t_inc_ = 1 min	2.00 × 10^−6^–1.00 × 10^−4^	4.50 × 10^−7^	Turmeric powder	[8]
LSV	Gr/GCE	~0.750 0.10 mol/L H_2_SO_4_ t_acc_ = 80 sec	5.00 × 10^−8^–3.00 × 10^−6^	3.00 × 10^−8^	*Curcuma longa* L.	[97]
DPV	erGO/GCE	~0.150 PBS pH 7.40 + 0.10 mol/L KCl	2.00 × 10^−7^–6.00 × 10^−5^	1.00 × 10^−7^	Turmeric capsules	[98]
CV	GO/GCE rGO/GCE	PBS pH 7.40	1.00 × 10^−12^–1.00 × 10^−9^ 1.00 × 10^−9^–1.00 × 10^−7^ 1.00 × 10^−12^–1.00 × 10^−10^ 1.00 × 10^−10^–1.00 × 10^−8^	9.00 × 10^−13^ 9.00 × 10^−13^		[108]
CV	Cu-GO/GCE Cu-rGO /GCE	PBS pH 7.40	-	4.70 × 10^−9^ 2.00 × 10^−11^	Plasma	[25]
DPV	pACBK/GCE	~0.175 PBS pH 6.40 t_acc_ = 70 sec	1.00 × 10^−7^–7.00 × 10^−5^	4.10 × 10^−8^	Human urine	[87]
SWV	Az-rGO@ MWCNTs /GCE	~0.450 PBS pH 3.50	8.00 × 10^−9^–2.00 × 10^−6^ 2.00 × 10^−6^–1.00 × 10^−5^	3.00 × 10^−9^	Curcuma tablets, human plasma, urine,	[105]
SDLSV	MnO_2_- c-MWCNTs /GCE	0.758 0.10 mol/L H_2_SO_4_ E_acc_ = −0.300 V; t_acc_ = 90 s	1.00 × 10^−8^–1.00 × 10^−6^ 1.00 × 10^−6^–8.00 × 10^−5^	6.00 × 10^−9^	Turmeric powder, curry, mustard, instant noodle seasoning, ginger powder	[19]
DPV	β-CD–rGO /GCE	~0.500 PBS pH 7.00 t_inc_ = 45 min	5.00 × 10^−8^–1.00 × 10^−5^	3.30 × 10^−8^	-	[46]
SWV	NSrGO/Ru@Au_NPs /GCE	~0.600 PBS pH 5.00	1.00 × 10^−12^–1.00 × 10^−10^	2.00 × 10^−13^	Plasma	[86]
CV	MBMIP_NPs /GCE	~0.150 PBS pH 3.06	1.00 × 10^−7^–1.00 × 10^−4^	1.00 × 10^−8^	Potato chips	[106]
DPV	pCys_MIP-CuCo_2_O_4_-*N*-CNTs-P-GO /GCE	~0.450 PBS pH 3.06	1.00 × 10^−7^–1.00 × 10^−6^ 1.00 × 10^−6^–3.00 × 10^−5^	3.00 × 10^−8^	Human blood serum	[76]
SWV	Al^3+^-Pd_NPs /PGE	0.560 PBS pH 2.00	3.00 × 10^−8^–6.00 × 10^−7^	2.20 × 10^−8^	Turmeric powder	[99]
SWV	Pd_NPs-pPr /PGE	0.504 PBS pH 2.00	5.00 × 10^−9^–1.00 × 10^−7^	1.20 × 10^−9^		[101]
DPV	HaP-IL/PGE	0.560 ABS pH 4.80 t_acc_ = 5 min	5.43 × 10^−6^–2.71 × 10^−5^	5.04 × 10^−6^	-	[109]
DPV	EPPGE	0.10 mol/L KCl pH 2.00	3.25 × 10^−7^–1.95 × 10^−6^	2.96 × 10^−7^	Turmeric powder	[16]
AdSCV	SPCE	0.700 0.10 mol/L HCl (40% ethanol); t_acc_ = 420 s	2.20 × 10^−6^–7.00 × 10^−5^	4.90 × 10^−6^	-	[110]
DPV	CPE	~0.650 PBS pH 3.00	3.00 × 10^−6^–3.00 × 10^−4^	5.03 × 10^−6^	Human blood serum	[104]
DPAdSV	CPE	0.300 ABS pH 3.50 E_acc_ = 0.800 V; t_acc_ = 120 s 0.600 ABS pH 3.50	5.76 × 10^−8^–4.83 × 10^−6^ 9.60 × 10^−7^–1.08 × 10^−5^			[20]
CV	CPE in 0.01 mol/L β-CD	0.800 ABS pH 3.57	2.50 × 10^−6^–2.70 × 10^−5^	9.30 × 10^−7^	Turmeric spice	[77]
DPV	rGO/CPE	~0.650 PBS pH 3.00	1.00 × 10^−5^–6.00 × 10^−3^	3.18 × 10^−6^	Human blood serum	[3]
CV	pTY/CPE	0.239 PBS pH 6.50	2.00 × 10^−6^–1.00 × 10^−5^ 1.00 × 10^−5^–4.00 × 10^−5^	1.09 × 10^−6^	Natural food supplement	[90]
DPV	pAA_MIP /GE	~0.700 ABS pH 5.50	1.00 × 10^−6^–1.00 × 10^−5^ 1.00 × 10^−5^–1.80 × 10^−4^	4.00 × 10^−8^	Raw turmeric, turmeric powder, capsule	[23]
CV	pMAA_MIP /CPE	0.434 PBS pH 3.06 t_acc_ = 20 s	1.00 × 10^−7^–5.00 × 10^−5^	1.01 × 10^−8^	Curcuma powder, cookies	[21]
CV	pTMS_MIP /CPE	0.400 and 0.700 PBS pH 6.00 t_acc_ = 20 s	1.00 × 10^−6^–1.00 × 10^−4^		Turmeric powder	[111]
SWV	CdO-IL/CPE	0.420 PBS pH 7.00	2.00 × 10^−7^–3.20 × 10^−4^	8.00 × 10^−8^	Spices	[96]
SWV	ZnO_NPs-PVP_NFs-FC /CPE	~0.250 PBS pH 8.00	1.00 × 10^−7^–7.00 × 10^−6^ 7.00 × 10^−6^–5.00 × 10^−4^	2.40 × 10^−8^	Urine; turmeric powder	[112]
DPV	SDS /CNTsPE	~0.150 PBS pH 6.00	2.00 × 10^−7^–1.00 × 10^−6^ 1.50 × 10^−6^–4.50 × 10^−6^	2.70 × 10^−8^	Natural food supplement	[18]
DPV	pGA /CNTsPE	0.116 PBS pH 7.50	4.00 × 10^−7^–6.00 × 10^−6^ 6.00 × 10^−6^–1.00 × 10^−5^	2.79 × 10^−8^	Food supplement	[89]
SWASV	Ce-BDC-MOF_NPs /GPE	~0.550 BRB pH 3.00 E_acc_ = 0.100 V; t_acc_ = 40 s	2.00 × 10^−11^–2.00 × 10^−9^ 2.00 × 10^−9^–9.00 × 10^−9^ 5.00 × 10^−11^–7.00 × 10^−9^ 3.00 × 10^−11^–6.00 × 10^−9^	6.00 × 10^−12^ 1.50 × 10^−11^ 9.00 × 10^−12^	Bulk Human plasma Human urine	[91]
DPV	p(Van-co-Caf)/Pt	~0.350 PBS pH 7.25	1.00 × 10^−5^–7.00 × 10^−5^ 1.00 × 10^−4^–1.00 × 10^−3^	5.00 × 10^−6^	Turmeric powder, curry powder	[6]
CV	CNTs-CMC /Au-PET	0.300 CAB pH 6.00	1.00 × 10^−6^–4.80 × 10^−6^	8.40 × 10^−8^	Turmeric powder	[100]
DPV	PACO_MIP /GCE	Indirect ~0.170 V 1.00 × 10^−3^ mol/L FCM ABS pH 6.50	1.00 × 10^−8^–2.00 × 10^−6^	5.00 × 10^−9^	Turmeric extract	[56]
PEC	TGACdTe@NiTAPc-Gr /ITO	PBS pH 8.00 E_appl_ = −0.250 V	2.50 × 10^−7^–1.00 × 10^−4^	1.25 × 10^−8^		[113]

**Table 2:** Analytical performances of CU-modified electrochemical sensors.

Technique	Electrode	Analyte	Linear Range (mol/L)	LOD (mol/L)	Sample	Ref.
LSV	polyCU/GCE	Epinephrine Paracetamol	4.90 × 10^−6^–2.31 × 10^−4^ 9.90 × 10^−7^–2.31 × 10^−4^	5.40 × 10^−8^ 1.10 × 10^−7^	Injections Tablets	[159]
Amp	CU-Ag_NPs/PWIGE	Paracetamol	5.90 × 10^−7^–3.42 × 10^−4^	2.90 × 10^−7^	−	[178]
CV	CU-CdSe_QDs/PWIGE	Ascorbic acid	1.86 × 10^−7^–7.00 × 10^−6^	−	−	[179]
CV	CU-chitosan/GCE	Bilirubin	1.00 × 10^−8^–1.00 × 10^−7^	3.30 × 10^−9^	Blood serum	[180]
DPV						
Amp	poly-GQDS/ITO	APO*e4* DNA	20.00–400.00 pg/mL	0.48 pg/mL	Human blood plasma	[174]
LSV	CU_QDs/GCE	Dopamine	5.00 × 10^−11^–1.00 × 10^−9^	6.00 × 10^−12^	−	[181]
LSV	polyCU-oCNTs/GCE	Dopamine Serotonin	1.00 × 10^−5^–1.70 × 10^−4^ 1.00 × 10^−5^–1.30 × 10^−4^	1.00 × 10^−5^ 1.10 × 10^−5^	−	[163]
DPV	CU-Ag_NPs-SDS-MWCNTs/GCE	Dopamine Guanine Uric acid	1.20 × 10^−5^–2.00 × 10^−4^ 1.60 × 10^−5^–4.00 × 10^−4^ 1.80 × 10^−5^–6.50 × 10^−4^	1.40 × 10^−7^ 1.90 × 10^−7^ 3.80 × 10^−7^	Blood, serum, urine, pharmaceuticals	[167]
EIS	poly(Ni^2+^-CU)/ Ni foam	Amyloid β oligomer	1.00 × 10^−12^–5.00 × 10^−9^	1.00 × 10^−12^	Artificial cerebrospinal fluid	[30]
Amp	poly(Ni^2+^-CU)/CPE	Amoxicillin	8.00 × 10^−6^–1.00 × 10^−4^	5.00 × 10^−6^	Urine, capsules	[142]
Amp	poly(Ni^2+^-CU)/GCE	NADH	3.00 × 10^−7^–3.00 × 10^−4^	1.80 × 10^−7^	Human serum	[182]
Amp	polyCU/PGE	Hydroxylamine	5.00 × 10^−7^–5.00 × 10^−4^	1.50 × 10^−7^	Pharmaceuticals water	[162]
Amp	Cu(II)-CU-SWCNTs/GCE	Hydroxylamine	1.00 × 10^−6^–1.00 × 10^−4^ 1.00 × 10^−4^–1.00 × 10^−3^	1.90 × 10^−8^	Pharmaceuticals	[151]
Amp	polyCU-MWCNTs/GCE	Hydrazine	2.00 × 10^−6^–4.40 × 10^−5^	1.40 × 10^−6^	−	[160]
DPV	MIpAA@CU/μPAD	Bisphenol A	4.38 × 10^−9^–8.76 × 10^−6^	2.06 × 10^−9^	Cans, plastic bottles	[173]
Amp FIA	polyCU-quinone-CB/GCE	Sulfide	1.00 × 10^−5^–1.00 × 10^−4^ 1.00 × 10^−5^–1.20 × 10^−3^	2.40 × 10^−6^ 7.12 × 10^−6^		[164]
CV	polyCU-MWCNTs/PWIGE	Butylated hydroxyanisole	3.37 × 10^−6^–3.32 × 10^−4^	2.25 × 10^−7^	−	[165]
DPV	CU-UiO-66/GCE	Methyl parathion	6.88 × 10^−8^–6.88 × 10^−5^	3.36 × 10^−9^	Vegetables, fruits	[171]
DPV	poly(Cu^2+^-CU)/GCE	4-Nitrophenol	1.00 × 10^−7^–1.03 × 10^−3^	6.82 × 10^−8^	−	[150]
LSV	Ni(CU)_2_-GO/GCE	4-Nitrophenol	4.90 × 10^−7^–7.60 × 10^−4^	1.60 × 10^−8^	−	[168]
DPV	CU_NPs-NiS_2_-rGO/SPCE	Methyl parathion 4-Nitrophenol	2.50 × 10^−6^–5.00 × 10^−6^ 5.00 × 10^−6^–8.00 × 10^−5^ 2.50 × 10^−6^–5.00 × 10^−6^ 5.00 × 10^−6^–8.00 × 10^−5^	8.70 × 10^−8^ 6.90 × 10^−8^	Tomato and apple juices; spiked river water	[166]
Amp	CU-Ag_NPs-rGO-FeCo_2_O_4_/SPCE	Hydrazine 4-Nitrophenol	2.00 × 10^−6^–3.00 × 10^−4^ 3.00 × 10^−4^–1.20 × 10^−3^ 2.00 × 10^−6^–3.00 × 10^−4^ 3.00 × 10^−4^–1.20 × 10^−3^	2.37 × 10^−8^ 1.84 × 10^−8^	Industrial wastewater, river water	[169]
DPV	MoS_2_-Au-polyCU/PGE	Hydrazine Nitrite	2.00 × 10^−5^–3.50 × 10^−4^ 3.50 × 10^−4^–1.20 × 10^−3^ 2.00 × 10^−5^–3.50 × 10^−4^ 3.50 × 10^−4^–1.20 × 10^−3^	1.83 × 10^−8^ 2.17 × 10^−8^	Industrial wastewater, river water	[161]
DPASV	polyCU_NSs/PWIGE	Hg^2+^	1.05 × 10^−9^–1.05 × 10^−7^	3.51 × 10^−10^	Seawater	[183]
DPV	polyCU-MnO_2_-Gr/GCE	Hg^2+^ CN^−^ F^−^	5.00 × 10^−8^–1.20 × 10^−6^ 5.00 × 10^−8^–1.20 × 10^−6^ 5.00 × 10^−8^–1.20 × 10^−6^	1.92 × 10^−8^ 2.83 × 10^−8^ 1.72 × 10^−8^	Spiked river water, tap water, petrochemical refinery wastewater	[170]
Potentiom	CU-CPE	Cu^2+^	1.00 × 10^−6^–1.00 × 10^−2^	1.00 × 10^−6^	Water	[184]

## Data Availability

No new data were created or analyzed in this study. Data sharing is not applicable to this article.

## Abbreviations

A	adenine;
ABS	acetate buffer solution;
ACV	alternating current voltammetry;
AdSCV	adsorptive stripping cyclic voltammetry;
AFB_1_	aflatoxin B_1_;
Al^3+^-Pd_NPs	aluminum ions/palladium nanoparticles;
Amp	amperometry;
AOA	antioxidant activity;
AOC	antioxidant capacity;
Au_NPs-FMWCNTs-IL-Chit	gold nanoparticles-amino functionalized multi-wall carbon nanotubes-ionic liquid-chitosan nanocomposite
Az-rGO@MWCNTs	azobenzene-modified reduced graphene oxide@multi-walled carbon nanotubes;
β-CD	beta-cyclodextrin;
β-CD–rGO	beta-cyclodextrin–reduced graphene oxide;
BDDE	boron doped diamond electrode;
BDMCU	bis-demethoxycurcumin;
BPPGE	basal plane pyrolytic graphite electrode;
BRB	Britton Robinson buffer;
C	cytosine;
CAB	citric acid buffer;
CB	carbon black;
CdO-IL	cadmium oxide-ionic liquid (1,3-dipropylimidazolium bromide);
Ce-BDC-MOF_NPs	Ce-1,4-benzenedicarboxylic metalorganic framework nanoparticles;
CNTs-CMC/Au-PET	carbon nanotubes-carboxymethylcellulose/Au on polyethylenetherephthalate;
CNTsPE	carbon nanotubes paste electrode;
CPE	carbon paste electrode;
CQDs	carbon quantum dots;
CTAB	cetyltrimethylammonium bromide;
ct-dsDNA	calf thymus double stranded DNA;
CU@ZIF-8-PDA	curcumin@zeolitic imidazolate framework-8-polydopamine;
CV	cyclic voltammetry;
DFT	density functional theory;
DMCU	demethoxycurcumin;
DME	dropping mercury electrode;
DMF	dimethylformamide;
DPP/DPV	differential pulse polarography/differential pulse voltammetry;
DPAd(C)SV	differential pulse adsorptive (cathodic) stripping voltammetry;
DPASV	differential pulse anodic stripping voltammetry;
DPPH	2,2-diphenyl-1-picrylhydrazyl;
Dy_NWs	dysprosium nanowires;
E_acc_	accumulation potential;
E_appl_	applied potential;
ECE	electrochemical-chemical-electrochemical;
EIS	electrochemical impedance spectroscopy;
E_p_ (E_pa_ and E_pc_)	peak potential (anodic peak potential and cathodic peak potential);
EPPGE	edge plane pyrolytic graphite electrode;
EQCM	electrochemical quartz crystal microbalance;
erGO	electrochemically reduced graphene oxide;
ET	electron transfer;
FCM	ferrocenemethanol;
FDA	US Food and Drug Administration
FFTSWV	fast Fourier transform square wave voltammetry;
FIA	flow injection analysis;
FRAP	ferric reducing antioxidant power;
GCE	glassy carbon electrode;
GE	graphite electrode;
GO	graphene oxide;
GPE	graphite paste electrode;
GQDs	graphene quantum dots;
Gr	graphene;
GRAS	Generally Recognized As Safe
HaP-IL	hydroxyapatite nanoparticles and ionic liquid;
HAT	H-atom transfer;
HER-2	human epidermal growth factor receptor 2
hIL-2	human interleukin-2;
HIV	human immunodeficiency virus;
HMDE	hanging mercury drop electrode;
HPSAM	H-point standard addition method;
HPV	human papilloma virus;
ITO	indium tin oxide;
LC-MS	Liquid chromatography-mass spectrometry
LOD	limit of detection;
LSV	linear sweep voltammetry;
MBA_pAAM	N, N’-methylenebisacrylamide cross-linked polyacrylamide;
MBMIP_NPs	magnetic biocompatibility molecularly imprinted nanoparticles;
MIP	molecularly imprinted polymer;
MIpAA@CU_NPs/μPAD	molecularly imprinted polyacrylamide coated curcumin nanoparticles micro-paper-based analytical device;
MnO_2_-c-MWCNTs	MnO_2_ nanoparticles functionalized carboxylated multi-walled carbon nanotubes;
MS	mass spectrometry;
MWCNTs	multi-walled carbon nanotubes;
NADH	β-nicotinamide adenine dinucleotide;
NOS	nitric oxide species;
NPs	nanoparticles;
NSs	nanospheres;
NSrGO/Ru@Au_NPs	Ru@Au nanoparticles decorated nitrogen and sulfur functionalized reduced graphene oxide;
oCNTs	oxidized carbon nanotubes;
pAA-MIP	polyacrylic-acid-based molecularly imprinted polymer;
pACBK	poly(acid chrome blue K);
PACO_MIP	4-pentenoyl-alanyl-chitosan oligosaccharide-based molecularly imprinted polymer;
PBS	phosphate buffer solution;
PC3	polyaniline–curcumin–copper–cobalt hybrid composite
PCR	polymerase chain reaction;
pCys_MIP-CuCo_2_O_4_-*N*-CNTs-P-GO	poly(L-cysteine)-based molecularly imprinted polymer-CuCo_2_O_4_-nitrogen-doped carbon nanotubes -phosphorus-doped graphene oxide;
Pd_NPs-pPr	palladium nanoparticles on polyproline film;
PEC	photoelectrochemistry;
pGA	poly(glutamine);
PGE	pencil graphite electrode;
pMAA_MIP	poly(methacrylic acid)-based molecularly imprinted polymer;
poly A	5′-AAA AAA AAA AAA AAA AAA-3′ where A: adenine;
poly C	5′-CCC CCC CCC CCC CCC CCC-3′ where C: cytosine;
poly G	5′-GGG GGG GGG GGG GGG GGG-3′ where G: guanine;
poly T	5′-TTT TTT TTT TTT TTT TTT-3′ where T: thymine;
potentiom	potentiometry;
PT	proton transfer;
pTY	poly(Titan yellow);
pTMS_MIP	poly(trimethoxysilane)-based molecularly imprinted polymer;
p(Van-co-Caf)	poly(vanillin-co-caffeic acid);
PWIGE	paraffin wax impregnated graphite electrode modified;
QDs	quantum dots;
R_ct_	charge transfer resistance at the electrode/solution interface;
rGO	reduced graphene oxide;
ROS	reactive oxygen species;
SASPM	surface active superparamagnetic maghemite (γ-Fe_2_O_3_);
SCE	saturated calomel electrode;
SDLSV	second-order derivative linear sweep voltammetry;
SDS	sodium dodecyl sulfate;
SPCE	screen printed carbon electrode;
SPE	screen printed electrode;
SWASV	square wave anodic stripping voltammetry;
SWV	square wave voltammetry;
T	thymine;
t_acc_	accumulation time;
TGACdTe@NiTAPc-Gr	thioglycolic acid-capped CdTe nanoparticles and nickel tetra-amined phthalocyanine-linked graphene oxide;
t_inc_	incubation time;
ZnO_NPs-PVP_NFs-FC	zinc oxide nanoparticles-poly vinyl pyrrolidone-nanofibers-ferrocene.
